# Analysis on the Physicochemical Properties of *Ginkgo biloba* Leaves after Enzymolysis Based Ultrasound Extraction and Soxhlet Extraction

**DOI:** 10.3390/molecules21010097

**Published:** 2016-01-15

**Authors:** Chang-Wei Zhang, Cheng-Zhang Wang, Ran Tao

**Affiliations:** 1 Institute of Chemical Industry of Forest Products, CAF, Nanjing 210042, Jiangsu, China; 13770752951@163.com (C.-W.Z.); trmoon1949@126.com (R.T.); 2 National Engineering Laboratory for Biomass Chemical Utilization, Nanjing 210042, Jiangsu, China; 3 Key and Open Laboratory on Forest Chemical Engineering, SFA, Nanjing 210042, Jiangsu, China; 4 Key Laboratory of Biomass Energy and Material, Nanjing 210042, Jiangsu, China; 5 Institute of New Technology of Forestry, CAF, Beijing 100091, China

**Keywords:** physicochemical properties, *Ginkgo biloba* leaves, enzymolysis, ultrasound, Soxhlet extraction

## Abstract

In this study, high performance liquid chromatography (HPLC), ultraviolet (UV), thermagravimetric analyzer (TGA), pyrolysis-gas chromatography-mass spectrometry (Py-GC-MS), and scanning electron microscope (SEM) were used as measurement techniques, contents of chemical composition, pyrolytic products, thermal stability, morphological characterization of *Ginkgo biloba* leaves (GBL) acted as the index, and physicochemical properties of GBL after enzymolysis based ultrasound extraction (EBUE) and Soxhlet extraction were studied. The detection results of chemical composition revealed that contents of general flavone, soluble protein, soluble total sugar and protein in the GBL declined significantly after EBUE, and contents of polyprenols and crude fat obviously reduced as well after Soxhlet extraction. Py-GC-MS results indicated that total GC contents of micromolecules with carbon less than 12 from 54.0% before EBUE decline to 8.34% after EBUE. Total GC contents of long-chain fatty acids with carbon less than 20 from 43.0% before EBUE reduced to 27.0% after Soxhlet extraction. Thermal stability results showed that GBL after Soxhlet extraction was easier to decompose than GBL before EBUE. SEM results illustrated that surface structure of GBL was damaged severely after EBUE, compared with GBL before EBUE, while organic solvent extraction had little influence on the morphological characterization of GBL after Soxhlet extraction compared with GBL after EBUE.

## 1. Introduction

*Ginkgo biloba* leaves (GBL) are a traditional Chinese medicine, which have survived more than 180 million years, and are considered to be “living fossils” [1,2]. GBL are rich in many biologically active compounds such as flavonoids, alkylphenols, carboxylic acids, terpene lactones, polyprenols and so on [3,4,5]. Polyprenols are especially important active ingredients in GBL. People have conducted much research on the polyprenols of GBL and found that they exhibited excellent biological activities such as antiviral, improving immunologic function, treating neurodegenerative diseases, cardiovascular diseases, memory disorders and so on [6,7,8,9,10]. Thus, researching about the extraction, isolation and purification of polyprenols from GBL has always been a hot spot, in which the extraction of polyprenols is one of the most important steps. To date, the main extraction method of polyprenols is organic solvent extraction, but its extraction efficiency is low. In order to increase extraction efficiency of polyprenols, many cell wall disruption technologies have been developed, in which enzymolysis-based ultrasound extraction (EBUE) is a relatively new cell wall disruption technology and was used in the extraction of many active substances [11], because it not only improves extraction efficiency but also contributes to the reduction of solvent, energy and waste [12,13]. However, few studies have been reported on the EBUE of GBL polyprenols.

Our group researched EBUE of polyprenols’ lipids from GBL in an earlier stage. First, ten kinds of enzymes were added into the enzymolysis system of GBL, respectively. Then, these enzymes were put into a water bath supersonic device for a certain time. After enzymolysis and ultrasound extraction and separating filtrate and filter residue, filter residue was dried at 50 °C, then put into Soxhlet extraction apparatus for Soxhlet extraction for about 9 h. Then, using the content of polyprenols in extracted GBL lipids as an index, we obtained optimum conditions of an enzyme quantity of 0.5 g (the mass ratio of cellulase and pectinase was 1:2, and the enzyme activity was 60 U/mg), enzymolysis pH of 4.5, ultrasonic power of 100 w, and temperature of ultrasound of 45 °C; under this condition, polyprenol yield increased by 69.70% compared with direct petroleum ether extraction. Then, we conducted a scale-up experiment on the basis of optimum conditions and found that the real and predicted results approached each other, thus, this optimum condition has the potential to be used for industrialization. However, most of researchers paid too much attention to the extraction of polyprenols from GBL, and systematic studies on physicochemical properties of GBL after extraction were often ignored. In fact, investigation into the physicochemical properties of GBL after extraction is helpful for exploring the reasons and mechanisms why extraction efficiency of polyprenols from GBL will increase; therefore, it is very meaningful.

With respect to analytic methods of physicochemical properties, there are lots of technologies such as high performance liquid chromatography (HPLC), ultraviolet (UV), thermagravimetric analyzer (TGA), pyrolysis-gas chromatography-mass spectrometry (Py-GC-MS), scanning electron microscope (SEM) and so on. HPLC and UV are generally applied to quantify chemical composition [14]. Py-GC-MS is an important technology that can provide compositional information of complex component macromolecules as well as characteristics of volatile pyrolysis products [15]. Moreover, it only requires a very small amount of samples and provides semi-quantitative results and information at a molecular level. TGA is always used to study bulk thermal decomposition and kinetic behavior of biomass at slow heating rates, while SEM is always used to observe the surface morphology and ultrastructure of sample.

In this study, physicochemical properties of GBL after EBUE and Soxhlet extraction were studied using HPLC, UV, Py-GC-MS, TGA and SEM as measurement techniques and utilizing GBL before EBUE as a blank control. The objective of this study is to probe into the difference of the contents of chemical composition, pyrolytic products, thermal stability, morphological characterisation of GBL before and after EBUE as well as after Soxhlet extraction, hoping to provide comprehensive understanding and theoretical guidance for reusing GBL after extraction.

## 2. Results and Discussion

### 2.1. Comparison of the Contents of Chemical Composition in GBL after EBUE and Soxhlet Extraction

Contents of polyprenols, general flavone, soluble total sugar, soluble protein, crude fat and protein in the GBL before and after EBUE as well as after Soxhlet extraction were shown in Table 1. From the table, we could conclude that contents of general flavone, soluble protein, soluble total sugar and protein in the GBL after EBUE declined significantly compared with GBL before EBUE. On the contrary, contents of polyprenols and crude fat in the GBL after EBUE increased compared with GBL before EBUE. Enzymes and ultrasounds could damage the cell wall of GBL, making more ingredients dissolve out, while ultrasounds could also increase degradations of fat and oils and promote some natural products to transform to other compounds [16,17]. Polyprenols and crude fat are water insoluble substances, while general flavone, soluble protein, soluble total sugar and protein are partially soluble or totally dissolve in water, with the removal of enzymatic hydrolysate from GBL, ingredients with the ability to be partially soluble or totally dissolve in water would run off from GBL, causing its contents to decline, but ingredients with water insolubility will remain in GBL, leading the content of polyprenols to increase. In addition, although ultrasounds might degrade part of the fat, disruption of the GBL cell wall would release more fat; hence, the content of crude fat also increased. After Soxhlet extraction, all of the contents of measured ingredients decreased compared with GBL after EBUE, in which contents of polyprenols and crude fat reduced significantly, while contents of other ingredients were almost invariant; the reason is that polyprenols and crude fat could dissolve into petroleum ether (Soxhlet extraction solvent), but other ingredients could not. With the separation between GBL and petroleum ether, contents of polyprenols and crude fat would decrease by a large margin, however, this action would have little influence on the substance content, which could not dissolve into petroleum ether.

**Table 1:** Contents of nutrition and active ingredients in the *Ginkgo biloba* leaves before and after enzymolysis based ultrasound extraction (EBUE).

Sample	Content/%					
	Polyprenols	General Flavone	Soluble Protein	Soluble Total Sugar	Crude Fat	Protein
GBL before EBUE	0.5274	0.6627	0.05205	16.52	8.192	10.35
GBL after EBUE	0.6285	0.1028	0.01106	4.289	8.659	8.966
GBL after Soxhlet extraction	0.05625	0.09856	0.01016	4.158	0.6862	8.758

### 2.2. Comparison of the Pyrolytic Products of GBL after EBUE and Soxhlet Extraction

**Figure 1:**
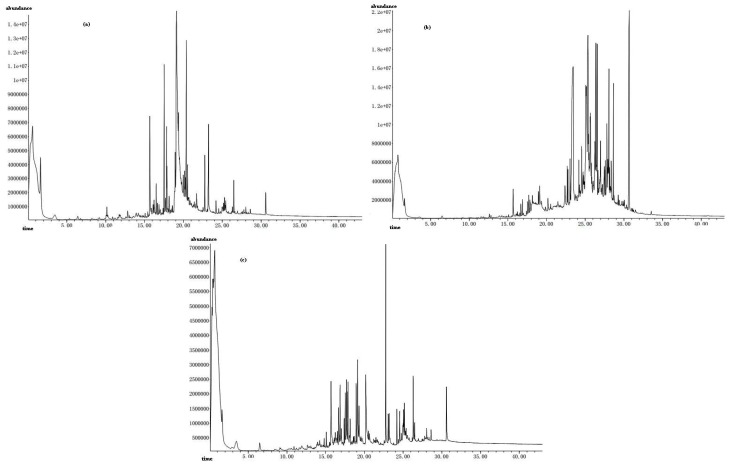
The total ion chromatogram of constituents after online pyrolytic methylation at 423 °C for GBL before (**a**) and after EBUE (**b**) as well as after Soxhlet extraction (**c**).

The total ion chromatogram of constituents after online pyrolytic methylation at 423 °C for GBL before and after EBUE as well as after Soxhlet extraction were shown in Figure 1a–c, respectively. Frow the Figure 1, we can see that peak number of GBL after EBUE and Soxhlet extraction were more than GBL before EBUE, this indicated that pyrolytic products of GBL after EBUE and Soxhlet extraction were more complex than GBL before EBUE.

Pyrolytic products of GBL before and after EBUE as well as after Soxhlet extraction were shown in Table 2. From the table, we could identify 20, 34 and 24 kinds of compounds before EBUE, after EBUE and after Soxhlet extraction, respectively. Pyrolytic products at the retention time of 0–22 min were mainly micromolecule compounds with carbon less than 12, in which 3-hydroxy-benzonic acid, 3-(4-hydroxyphenyl)-2-crylic acid, 3,4-dihydroxy-phenylacetic acid, 4-hydroxy-benzonic acid, *p*-hydroxycinnamic acid and d-quininic acid are phenolic acid compounds; 3,4,5-trihydroxy-methylbenzene, 2,4,6-trihydroxy-methylbenzene, 4-hydroxy-styrene, hydroquinone, trihydroxybenneze, 1,2,4-hydroxy-hydroquinone and 3,5-dihydroxylbenzamide are phenol compounds; 1,6-anhydro-β-d-glucose is a monose compound; and inositol is vitamin compound.

Pyrolytic products at the retention time of 22–26 min were mainly long-chain fatty acid compounds with carbon less than 20, in which myristic acid, 14-methyl-17-daturic acid, palmitic acid and stearic acid are saturated fatty acid compounds; trans-9-octadecenoic acid, trans-oleic acid, oleic acid are unsaturated fatty acid compounds. Pyrolytic products at the retention time of 26–31 min were mainly alkyl acid, esters, ketone compounds, and so on, in which docosanoic acid is alkyl acid; phthalic acid (2-ethylhexyl) monoester, terephthalic-2-ethylhexyl-essien ester and phthalic acid-dioctyl phthalate are ester compounds; 1,2,4-trihydroxy-9,10-amerantrone and boldenone are ketone compounds.

In addition, we also found total GC contents of micromolecules with carbon less than 12 from 54.0% before EBUE decline to 8.34% after EBUE, while reaching up to 67.3% after Soxhlet extraction; Total GC contents of long-chain fatty acids with carbon less than 20 from 43.0% before EBUE increase to 70.9% after EBUE, while reducing to 27.0% after Soxhlet extraction. Total GC content of alkyl acid compounds and so on with carbon more than 20 in GBL from 3.0% before EBUE increased to 20.8% after EBUE and changed to 5.67% after Soxhlet extraction. Corresponding contents of chemical composition in GBL varied from different extraction processes, and these chemical compositions contained micromolecule compounds and macromolecule organics. Most of the micromolecule compounds were water-soluble and would dissolve into enzymatic hydrolysate in the process of EBUE. After EBUE and with the removal of enzymatic hydrolysate from GBL, the contents of these substances would decrease significantly. Macromolecule organics are able to dissolve into organic solvents, and, in this study, petroleum ethyl was used as the extraction solvent in Soxhlet extraction. Therefore, some polar ingredients in GBL would dissolve into it and run off with separation between GBL and extraction solvent, causing total GC contents of macromolecule organics to decline after Soxhlet extraction.

### 2.3. Comparision of the Thermal Stability of GBL after EBUE and Soxhlet Extraction

**Figure 2:**
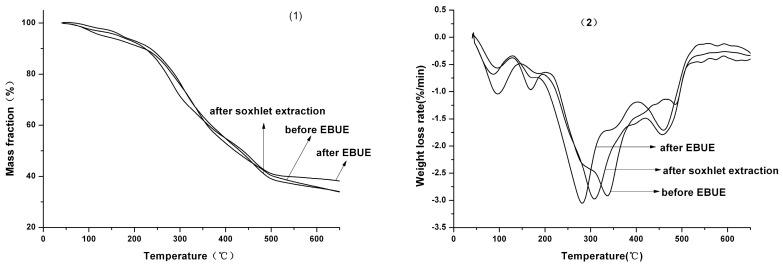
TG (**1**) and DTG (**2**) curves of GBL before and after EBUE as well as Soxhlet extraction.

TG and DTG curves of GBL before and after EBUE as well as after Soxhlet extraction were shown in Figure 2. In addition, characteristic parameters in thermal decomposition of GBL before and after EBUE as well as Soxhlet extraction were shown in Table 3. From the figure, we can conclude that GBL was dried by unbound water of physical absorption and bound water of chemical action between 41 °C and 151 °C, and a weight loss rate of 5.857%. As shown in the table, GBL mainly conducted thermal decomposition of three stages after 151 °C. The main ingredients of GBL consist of cellulose, hemicellulose and lignin, in which hemicellulose is the most unstable (decomposition temperature is about 225–325 °C), and cellulose takes second place (decomposition temperature is about 300–375 °C), while the decomposition temperature of lignin has the longest span (decompose step by step at 250–500 °C) [18].

**Table 2:** Pyrolytic products for *Ginkgo biloba* leaves (GBL) before and after EBUE as well as after Soxhlet extraction.

No.	Retention Time/Min	Molecular Formula	Chemical Name	Original Molecular Formula	Original Chemical Name	GC Content/%		
						Before EBUE	After EBUE	After Soxhlet Extraction
1	6.479	C_6_H_14_O_3_	1,2,3-trimethoxypropane	C_3_H_8_O_3_	glycerol	-	-	0.41
2	10.158	C_10_H_16_	dipentene	C_10_H_16_	-	0.25	-	-
3	12.595	C_9_H_10_O	4-methoxystyrene	C_8_H_8_O	4-hydroxy-styrene	-	0.08	-
4	12.824	C_8_H_10_O_2_	hydroquinone Dimethyl	C_6_H_6_O_2_	hydroquinone	0.34	-	-
5	15.685	C_9_H_10_O_3_	3-methoxy-benzonic acid-methyl ester	C_7_H_6_O_3_	3-hydroxy-benzonic acid	3.59	0.46	2.43
6	16.109	C_9_H_12_O_3_	1,2,4-trimethoxybenzene	C_6_H_6_O_3_	1,2,4-hydroxy-hydroquinone	0.81	-	0.32
7	16.223	C_9_H_10_O_3_	4-methoxy-benzonic acid-methyl ester	C_7_H_6_O_3_	4-hydroxy-benzonic acid	0.71	0.08	0.55
8	16.498	C_9_H_16_O_8_	1,6-anhydro-β-d-glucose-glyceryl polyther	C_6_H_10_O_5_	1,6-anhydro-β-d-glucose	1.12	-	-
9	16.538	C_10_H_14_O_3_	3,4,5-trimethoxy-methylbenzene	C_7_H_8_O_3_	3,4,5-trihydroxy-methylbenzene	-	-	0.44
10	16.669	C_9_H_12_O_3_	1,3,5-trimethoxy-benzene	C_6_H_6_O_3_	m-trihydroxybenneze	0.45	0.20	1.10
11	17.528	C_12_H_24_O_6_	1,2,3,4,5,6-hexa-methoxy-inositol	C_6_H_12_O_6_	inositol	1.67	-	1.78
12	17.694	C_10_H_14_O_3_	2,4,6-trimethoxy-methylbenzene	C_7_H_8_O_3_	2,4,6-trihydroxy-methylbenzene	0.79	0.29	1.87
13	17.888	C_13_H_26_O_7_	2,4,5,6,7-pentamethoxy-heptylic acid-methyl ester	C_7_H_14_O_7_	2,4,5,6,7-penta hydroxy-heptylic acid	0.98	0.12	1.15
14	18.163	C_14_H_12_O	1,2-dimethyl-methyl naphthol-furan	C_14_H_12_O	-	-	-	0.49
15	18.169	C_10_H_12_O_4_	3,4-dimethoxy-phenylacetic acid	C_8_H_8_O_4_	3,4-dihydroxy-phenylacetic acid	-	0.41	-
16	18.941	C_12_H_22_O_6_	d-quininic acid-tetramethyl-methyl ester	C_7_H_12_O_6_	d-quininic acid	1.78	0.19	0.87
17	19.101	C_10_H_10_O_3_	*p*-methoxycinnamic acid	C_9_H_8_O_3_	*p*-hydroxycinnamic acid	-	0.38	0.32
18	20.171	C_11_H_12_O_3_	*p*-methoxycinnamic acid-methyl ester	C_9_H_8_O_3_	*p*-hydroxycinnamic acid	0.97	0.28	2.51
19	20.171	C_11_H_12_O_3_	2-crylic acid-3-(4-methoxyphenyl) methyl ester	C_9_H_8_O_3_	3-(4-hydroxyphenyl)-2-crylic acid	-	-	3.23
20	20.372	C_12_H_16_O_4_	Phenethyl alcohol	C_12_H_16_O_4_	-	4.20	-	-
21	20.538	C_15_H_30_O_2_	tetradecanoic acid-methyl ester	C_14_H_28_O_2_	myristic acid	0.63	0.07	0.26
22	21.459	C_9_H_11_NO_3_	3,5-dimethoxybenzamide	C_7_H_7_NO_3_	3,5-dihydroxylbenzamide	-	0.20	-
23	22.374	C_12_H_14_O_4_	2-crylic acid-3-(3,4-dimethoxyphenyl)methyl ester	C_10_H_10_O_4_	3-(3,4-dihydroxyphenyl)-2-crylic acid	-	0.78	-
24	22.666	C_17_H_34_O_2_	palmitic acid-methyl ester	C_16_H_32_O_2_	palmitic acid	1.17	1.07	3.14
25	23.439	C_16_H_32_O_2_	palmitic acid	C_16_H_32_O_2_	-	3.26	12.4	0.56
26	24.251	C_17_H_34_O_2_	heptadecanoic acid	C_17_H_34_O_2_	-	-	0.57	-
27	24.531	C_19_H_38_O_2_	trans-9-octadecenoic acid-methyl ester	C_18_H_36_O_2_	trans-9-octadecenoic acid	-	1.78	-
28	24.543	C_19_H_36_O_2_	trans-oleic acid-methyl ester	C_18_H_34_O_2_	trans-oleic acid	-	-	0.56
29	24.772	C_19_H_38_O_2_	daturic acid-14-methyl-methyl ester	C_18_H_36_O_2_	14-methyl-17-daturic acid	-	0.61	-
30	24.772	C_19_H_38_O_2_	stearic acid-methyl ester	C_18_H_36_O_2_	stearic acid	1.17	-	0.15
31	24.852	C_15_H_11_O_3_	1-hydroxy-2-methyanthraquinone	C_15_H_11_O_3_	-	-	0.71	-
32	25.007	C_18_H_34_O_2_	oleic acid	C_18_H_34_O_2_	-	-	-	0.55
33	25.058	C_19_H_36_O_2_	oleic acid-methyl ester	C_18_H_34_O_2_	oleic acid	-	-	0.58
34	25.075	C_18_H_34_O_2_	octadecenoic acid-methyl ester	C_18_H_34_O_2_	-	-	3.59	-
35	25.149	C_18_H_34_O_2_	trans-13-octadecenoic acid	C_18_H_34_O_2_	-	0.04	4.88	-
36	25.167	C_18_H_36_O_2_	stearic acid	C_18_H_36_O_2_	-	0.24	3.59	0.83
37	25.470	C_20_H_40_O_2_	11-octadecenoic acid-isopropyl ester	C_20_H_40_O_2_	-	-	0.62	-
38	25.516	C_18_H_32_O_2_	linoleic acid	C_18_H_32_O_2_	-	-	0.75	-
39	25.790	C_20_H_34_O	tridecylanisole	C_19_H_32_O	tridecylphenol	-	0.71	-
40	26.540	C_19_H_36_O_3_	10-oxo-stearic acid-methyl ester	C_18_H_34_O_3_	10-oxo-stearic acid	-		-
41	26.654	C_21_H_42_O_2_	arachidic acid-methyl ester	C_20_H_40_O_2_	arachidic acid	-	0.65	-
42	28.136	C_17_H_32_O	(*Z*)-14-methyl-8-hexadecenal	C_17_H_32_O	-	-	0.60	-
43	28.234	C_18_H_34_O_3_	trans-9,10-epoxyoctadecanoic acid	C_18_H_34_O_3_	-	-	0.37	-
44	28.354	C_23_H_46_O_2_	docosanoic acid-methyl ester	C_22_H_44_O_2_	docosanoic acid	-	0.80	-
45	28.634	C_16_H_22_O_4_	phthalic acid (2-ethylhexyl)monoester	C_16_H_22_O_4_	-	-	1.55	0.18
46	28.720	C_15_H_10_O_5_	2,4-dihydroxyl-1-methoxy-9,10-amerantrone	C_14_H_8_O_5_	1,2,4-trihydroxy-9,10-amerantrone	-	0.14	-
47	29.407	C_19_H_26_O_2_	boldenone	C_19_H_26_O_2_	-	-	0.08	-
48	29.744	C_14_H_24_	dispiro-tetradecane	C_14_H_24_	-	-	0.13	-
49	29.962	C_15_H_13_N	1-methyl-2phenyl-benzpyrole	C_15_H_13_N	-	-	0.08	-
50	30.585	C_24_H_38_O_4_	phthalic acid-dioctyl phthalate	C_24_H_38_O_4_	-	0.74		1.43
51	30.688	C_22_H_36_O_6_	terephthalic-2-ethylhexyl-essien ester	C_22_H_36_O_6_	-	-	6.74	-

**Table 3:** Characteristic parameters in thermal decomposition of GBL before and after EBUE as well as Soxhlet extraction.

Sample	Steps	Start Temperature/°C	End Temperature/°C	Weight Loss Percent/%	Remaining Percent/%
GBL before EBUE	1	151	201	2.880	91.26
	2	201	466	46.85	44.41
	3	466	591	8.130	36.28
GBL after EBUE	1	128	181	2.750	93.91
	2	181	403	39.39	54.52
	3	403	558	14.81	39.71
GBL after Soxhlet extraction	1	128	195	4.390	93.35
	2	195	420	41.67	51.68
	3	420	593	15.92	35.76

Therefore, GBL conducted decomposition of the first stage at 151–201 °C, which mainly included decomposition of part of the micromolecules as well as a bit of the hemicellulose in GBL. The decomposition of those substances are able to produce lots of micromolecular volatile gas, resulting in weight loss rate of 2.870%. Decomposition of the second stage for GBL was conducted at 201–466 °C, most of the micromolecule compounds and cellulose as well as the remaining hemicellulose generally completed decomposition during this stage. Moreover, with partial decomposition of lignin, a great deal of micromolecular volatile gas and condensable volatility macromolecule were produced [19], leading to weight loss rate of 46.86%; Decomposition of the third stage for GBL was conducted at 466–591 °C, mainly consisting of decomposition of remaining lignin and heavy hydrocarbon. Lignin is a copolymer composed by phenyl propane monomer [20] and is hard to decompose, which is similar to heavy hydrocarbon, so decomposition temperature of this stage is higher. At this time, the curve value of DTG started to decrease, indicating that the speed rate of decomposition for GBL became slow, and solid carbon particle, tar and hydrogen would be produced, causing a weight loss rate of 8.130%.

From the table and figure, we also could see that the decomposition process of GBL before and after EBUE as well as after Soxhlet extraction were similar. Only the range of decomposition temperature for each other were different. Decomposition temperature of GBL before EBUE was 23 °C higher than GBL after EBUE and after Soxhlet extraction. From the beginning of decomposition temperature, the speed rate of decomposition for GBL before EBUE was lower than GBL after EBUE at the temperature of 138–291 °C and 421–473 °C, while the results were in contrast at the temperature of 291–421 °C and more than 473 °C. The speed rate of decomposition for GBL before EBUE was lower than GBL after Soxhlet extraction at the temperature of 141–321 °C and more than 381 °C, while the results were in the contrast at the temperature of 321–381 °C. Moreover, the remaining percent of GBL before EBUE were higher than GBL after Soxhlet extraction at the end of decomposition for GBL. All of these results indicated that GBL after Soxhlet extraction was easier to decompose than GBL before EBUE. In addition, the speed rate of decomposition for GBL after EBUE was lower than GBL after Soxhlet extraction at the temperature of 151–288 °C, while the results were in the contrast at the temperature of more than 288 °C.

### 2.4. Comparision of Morphological Characterization of GBL after EBUE and Soxhlet Extraction

**Figure 3:**
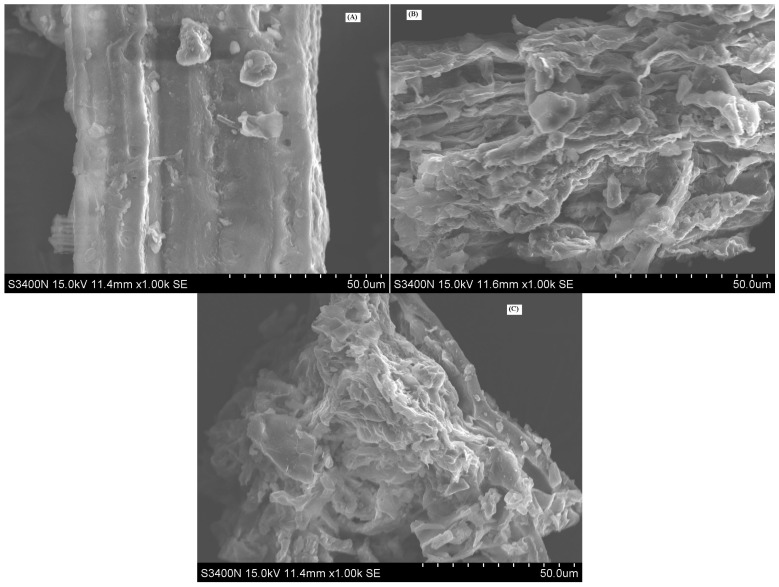
SEM pictures of GBL before (**A**) and after (**B**) EBUE as well as Soxhlet extraction (**C**).

The results of SEM pictures for GBL before and after EBUE as well as Soxhlet extraction were shown in Figure 3. As shown in the figure, surface structure of GBL before EBUE was smooth and the adjacent holes of the cell wall arranged densely. After EBUE, surface structure of GBL was damaged severely, lots of holes appeared and adjacent holes were loosely arranged. This is because of the combined action of enzymolysis and ultrasounds. Ultrasounds use their cavitation effects to crack cell walls and membranes [21,22], while enzymolysis can effectively catalyze the degradation of the cell wall [23,24], resulting in the structure of cell wall being changed. The change of cell wall for GBL in favor of solvent permeating into the interior of the cell urged some ingredients to combine with the cell wall or be located at the cell interior to release into the exterior, increasing the effect of extraction. After Soxhlet extraction, surface structure of GBL was almost invariant compared with GBL after EBUE. This illustrated that Soxhlet extraction had little influence on the structure of GBL.

## 3. Materials and Methods

### 3.1. Chemicals and Reagents

The dried GBL before and after EBUE as well as after Soxhlet extraction were prepared at the Institute of Chemical Industry of Forest Products (Jiangsu, China). The standard polyprenols (C_70_, C_75_–C_105_, C_110_, C_115_, C_120_) were purchased from Larodan Fine Chemical Co., Ltd. (Malmö, Sweden). Rutin, bovine serum albumin (BSA), tetramethylammonium (TMAH), coomassie brilliant blue (CBB), copper sulfate, potassium sulphate, boric acid, anthranone, sulfuric acid, phosphoric acid, sodium hydroxide, methyl red, methylene blue, ethanol, petroleum ether, and normal hexane were all purchased from Aladdin Chemicals (Shanghai, China).

### 3.2. Detection Methods of Chemical Composition for GBL

In order to compare the difference of the contents of chemical composition in GBL before and after EBUE as well as after Soxhlet extraction, contents of polyprenols, general flavones, soluble total sugar, crude fat, soluble protein, and protein were measured. The detection of polyprenols referred to high performance liquid chromatography. The detection of general flavones and soluble total sugar referred to ultraviolet spectrophotometry. The detection of crude fat referred to the method of Soxhlet extraction. The detection of soluble protein referred to the Coomassie brilliant blue method. The detection of protein referred to the Kjeldahl method.

### 3.3. Identification of Pyrolytic Products for GBL Using Py-GC-MC

Py-GC-MS was performed using a CDS 2000 pyroprobe, coupled to a Thermo Finnigan Focus DSQ GC–MS equipped with a J & W DB-1MS column (30 m × 0.25 mm i.d. × 0.25 μm film thickness, J & W Scientific, California, America). The pyrolyzer consists of a Pt filament and a control unit. Chromatographic separation was achieved by using an Agilent HP-5 column (30 m × 0.25 mm). The column was held at 50 °C for 5 min followed by a ramped temperature increase to 280 °C at a rate of 10 °C/min. The GC oven temperature was 300 °C, and the carrier gas was helium. The injector’s and MSDs’ transfer line temperature were held at 280 °C, while the ion source at 200 °C. A split ratio of 20:1 was used and the MS scan ranged between 28 and 500 *m*/*z*. Macroscopic GBL before and after EBUE as well as after Soxhlet extraction were put into the cracker, and pyrolysis was performed for 5 s at 423 °C. The chromatograms gathered were qualitatively analyzed using Nist02 library.

### 3.4. Analysis on the Thermal Stability of GBL Using TGA

Thermal decomposition of the GBL before and after EBUE as well as after Soxhlet extraction was compared by thermogravimetric using a thermogravimetric analyzer (STA409C/PC, Berlin, Germany). A 10 mg sample in a covered alumina crucible was pyrolyzed from room temperature to 650 °C at a constant thermaling rate of 10 °C/min. To maintain an inert atmosphere during pyrolysis, the purified nitrogen carrier gas was made to flow at a rate of 50 mL/min.

### 3.5. Morphological Characterization Observation of GBL Using SEM

Scanning electron microscope was used to observe the distinction among the GBL before and after EBUE as well as after Soxhlet extraction. The dried GBL samples were mounted on an SEM stub withdouble-sided adhesive tape and coated with a 50~100 nm thickness gold layer. Granule morphologies of GBL before and after EBUE as well as after Soxhlet extraction were examined in an SEM (S-3400N, Hitachi Limited, Tokyo, Japan) at an acceleration voltage of 20 keV and 100× magnification, respectively.

## 4. Conclusions

Our study revealed that contents of general flavones, soluble protein, soluble total sugar, and protein in the GBL after EBUE declined significantly, and contents of polyprenols and crude fat obviously reduced as well after Soxhlet extraction. Py-GC-MS results indicated that enzymolysis and ultrasounds could damage the cell wall of GBL, making more ingredients dissolve out. Thermal stability results showed that GBL after Soxhlet extraction was easier to decompose than GBL before EBUE. SEM results illustrated that the surface structure of GBL was damaged severely after EBUE, while surface structure of GBL was almost invariant after Soxhlet extraction. In conclusion, all of those results proved that both enzymolysis based ultrasound extraction and Soxhlet extraction wounds have certain influence on contents of related ingredients, thermal stability, morphological characterization and pyrolytic products of GBL. We hope these experimental results can provide reference for a comprehensive understanding of GBL after different extraction processes.

## Abbreviations

The following abbreviations are used in this manuscript:

GBL: *Ginkgo biloba* leaves
EBUE: enzymolysis based ultrasound extraction
